# The Complexity of Malignant Glioma Treatment

**DOI:** 10.3390/cancers17050879

**Published:** 2025-03-04

**Authors:** Linde F. C. Kampers, Dennis S. Metselaar, Maria Vinci, Fabio Scirocchi, Sophie Veldhuijzen van Zanten, Matthias Eyrich, Veronica Biassoni, Esther Hulleman, Michael Karremann, Wilfried Stücker, Stefaan W. Van Gool

**Affiliations:** 1 Immun-Onkologisches Zentrum Köln, 50674 Köln, Germany; l.kampers@iozk-lab.de (L.F.C.K.); stuecker@iozk.de (W.S.); 2 Princess Máxima Center for Pediatric Oncology, 3584 CS Utrecht, The Netherlands; d.s.metselaar@prinsesmaximacentrum.nl (D.S.M.); s.veldhuijzenvanzanten@erasmusmc.nl (S.V.v.Z.); e.hulleman@prinsesmaximacentrum.nl (E.H.); 3 Hopp Children’s Cancer Center Heidelberg (KiTZ), 69120 Heidelberg, Germany; 4 German Cancer Research Center (DKFZ), 69120 Heidelberg, Germany; 5 German Consortium (DKTK), 69120 Heidelberg, Germany; 6 Bambino Gesu’ Children’s Hospital-IRCCS, 00165 Rome, RM, Italy; maria.vinci@opbg.net (M.V.); fabio.scirocchi@opbg.net (F.S.); 7 Department of Radiology & Nuclear Medicine, Erasmus MC—University Medical Centre Rotterdam, 3015 GD Rotterdam, The Netherlands; 8 Brain Tumour Centre, Erasmus MC Cancer Institute, 3015 GD Rotterdam, The Netherlands; 9 University Hospital Würzburg, 97080 Würzburg, Germany; eyrich_m@ukw.de; 10 Fondazione IRCCS Istituto Nazionale dei Tumori, 20133 Milano, MI, Italy; veronica.biassoni@istitutotumori.mi.it; 11 University Hospital Mannheim, 68166 Mannheim, Germany; michael.karremann@umm.de

**Keywords:** tumor microenvironment, malignant glioma, immunotherapy

## Abstract

Malignant glioma is a highly aggressive, therapeutically non-responsive, and deadly disease with a unique tumor microenvironment (TME). Of the 14 currently recognized and described cancer hallmarks, five are especially implicated in malignant glioma and targetable with repurposed drugs: cancer stem-like cells, in general, and glioma stem-like cells in particular (GSCs), vascularization and hypoxia, metabolic reprogramming, tumor-promoting inflammation and sustained proliferative signaling. Each hallmark drives malignant glioma development, both individually and through interactions with other hallmarks, in which the TME plays a critical role. To combat the aggressive malignant glioma spatio-temporal heterogeneity driven by TME interactions, and to overcome its therapeutic challenges, a combined treatment strategy including anticancer therapies, repurposed drugs and multimodal immunotherapy should be the aim for future treatment approaches.

## Simple Summary

This review describes the dynamic influence of the tumor microenvironment during treatment of malignant glioma. The mechanism behind five hallmarks are outlined: glioma stem-like cells in particular (GSCs), vascularization and hypoxia, metabolic reprogramming, tumor-promoting inflammation and sustained proliferative signaling. A multimodal immunotherapy treatment plan is proposed, explaining how each hallmark can be targeted over time. Repeated tumor monitoring is deemed vital to alter the treatment plan when needed.

## 1. The Impactful Tumor Microenvironment

**Figure 1:**
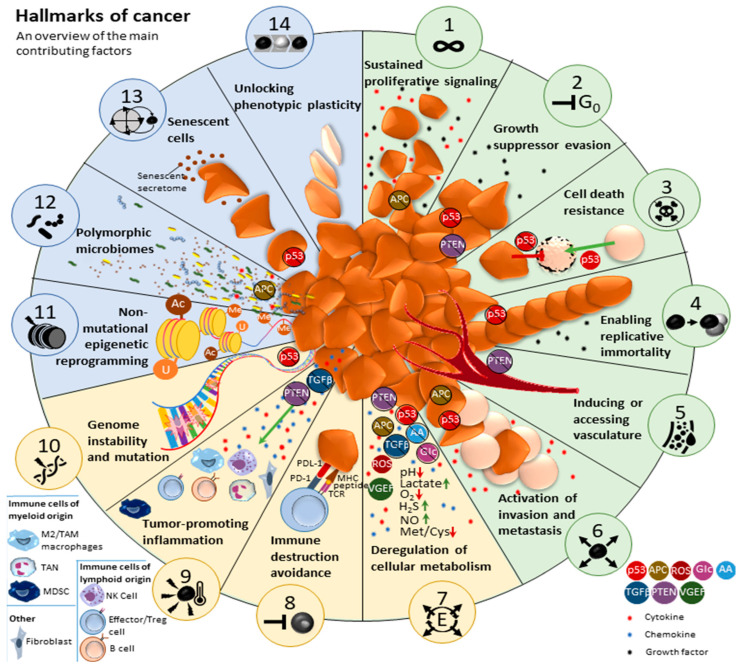
Top: An overview of the main contributing factors to the 14 hallmarks of cancer [1,2,3]. In green, the first hallmarks described. In yellow, hallmarks recognized and added in 2011. In blue, four additional hallmarks added in 2022. Legends on the lower left and right side indicate cell types, cytokines, chemokines, growth factors, and other factors vital in tumor development. Dark brown cells of irregular shapes represent tumor cells. Each hallmark has been given an icon, indicated by the numbered images in circles at the end of each hallmark pie slice. These icons will be used to indicate each hallmark in further figures.

The significant advancements in cancer biology is reflected in the discovery and description of the 14 hallmarks of cancer [1,2,3] (Figure 1). In 2000, the first 6 hallmarks were summarized [1]: (i) acquired capabilities for sustaining proliferative signaling, (ii) evading growth suppressors, (iii) resisting cell death, (iv) enabling replicative immortality, (v) inducing or accessing vasculature, and (vi) activating invasion and metastasis. In 2011 [2], four novel hallmarks were defined: (vii) deregulating cellular metabolism, (viii) avoiding immune destruction, (ix) tumor-promoting inflammation, and (x) genome instability and mutation. These latter hallmarks marked the recognition that a tumor process is not only characterized by intrinsic tumor cell characteristics but also by its specific tumor microenvironment (TME). In 2022 [3], four additional hallmarks were included: (xi) non-mutational epigenetic reprogramming, (xii) polymorphic microbiomes, (xiii) senescent cells, and (xiv) unlocking phenotypic plasticity (Figure 1). Again, these added hallmarks refer to the influence of the TME on tumor cell biology.

Clearly, the role of the TME should not be underestimated. As the source of immune cells, nutrients, oxygen, extracellular communicative signals, polymorphic microbes, and as the boundary between the tumor and the rest of the body, the TME must be prioritized in ongoing and future research. Particularly, the difference in TME between individual patients, its temporal plasticity, and how it is affected by different treatment options, should be considered in future anticancer treatments.

The categorization of tumors of the central nervous system (CNS) is changing, as it has over decades, based on gained insights in tumor biology and pathology [4]. Glioblastoma (GBM), IDH-wildtype, belongs to the category of adult-type diffuse gliomas and is a highly aggressive and infiltrative, mutative, therapeutically non-responsive, complex located, and deadly disease, which make it difficult to treat. GBM is the most frequent primary brain tumor in adults. Despite the highest mean years of potential life lost amongst all human cancers [5], the standard of care has not improved over the last two decades. Therefore, our primary focus in this narrative review will be on the complexity of GBM treatment, which will then be compared to other malignant gliomas in terms of heterogeneity, development, and treatment upon progression or relapse.

GBM has a diverse TME, consisting of heterogeneous and interactive populations of cancer cells and cancer stem-like cells, along with recruited stromal cell types, transformed parenchyma and associated stroma [3]. As well as the tumor cells, the TME includes the following different cell populations: (1) endothelial cells, (2) cells of lymphoid origin like effector T cells, regulatory T cells (Tregs), B cells and Natural Killer (NK) cells, (3) mesenchymal cells, (4) immune cells of myeloid origin, such as tumor-associated macrophages and microglia (TAMs, also known as glioma-associated macrophages or microglia, or GAMs), M2 macrophages, myeloid-derived suppressor cells (MDSC), tumor-associated neutrophils (TAN), and (5) fibroblasts [6]. Unique to the GBM environment are the neurons, glia, and microglia. Tumor cells communicate continuously with each other and with the TME via different chemical and electrochemical stimuli. The tumor can reprogram the function of the TME, resulting in an immunosuppressive and inflammatory environment in which cancer can thrive. The blood–brain barrier (BBB) limits the outflow of circulating tumor cells and thus aids in inhibiting extracranial metastasis, though this also complicates tumor analysis via non-invasive methods and limits treatment influx via systemic administration. The uniqueness of the CNS TME might further limit extracranial metastases to develop.

Here, we first outline the current Standard-of-Care (SoC) and tumor monitoring towards GBM treatment. Then, we zoom in on the most relevant GBM cancer-associated hallmarks, especially those implicated in GBM development, their spatiotemporal evolution, and their interplay with each other and the surrounding TME. These five hallmarks are deemed vital to understanding treatment mechanisms, all have currently available treatment options with repurposed drugs and are focus of current clinical research. We then compare adult and pediatric-type diffuse high-grade gliomas (diffuse pediatric-type high-grade glioma H3-wildtype and IDH-wildtype, diffuse midline glioma H3 K27-altered, and diffuse hemispheric glioma H3G34-mutant) to describe necessary treatment adaptations. Finally, we outline an empirically developed multiphase individualized combination treatment strategy to combat malignant glioma.

## 2. The Influence of the GBM TME on Prognosis

Currently, GBM is treated with standard of care (SoC): maximal safe resection, radiochemotherapy (RCT) and, in most cases, maintenance chemotherapy (CTx) [7,8], though GBM displays high treatment-resistance [9].

GBM tumor cells show plasticity and dynamic changes in response to tumor development and treatment, which are in part influenced by epigenetic pathways [10]. As the tumor changes and evolves over time, the TME composition shows a similar spatio-temporal heterogeneity and adaptation. TME cells, such as myeloid cells, are interwoven throughout the tumor. Moreover, intra- and extratumoral communication paths exist through the formation of heterogeneous and cooperative tumor functional networks which contribute to tumor growth and therapy resistance [11,12]. Many GBM cells build ultra-long membrane protrusions, or tumor microtubes: direct, long-distance (>500 μm) cell-to-cell connections used for brain invasion, proliferation, and therapy resistance. Cells interconnected via tumor microtubes are shown to be protected from cell death by RT. When damage to the tumor occurs, tumor microtubes are used for repair [13]. These interconnections occur through the formation of tumor microtubes [13], through autocrine/paracrine signaling, the release and uptake of exosomes [14], as well as through glioma-neuronal active synaptic activity [15,16,17].

Rather than one dense mass of tumor, different strictly regulated tumoral developmental areas exist. A tumor functions as a well-oiled machine, similar to a complex organ, a fact often disregarded both in clinical treatment as well as in general discussions [6]. The tumor and TME heterogeneity lead to strong patient-specific differences in anticancer treatment response. As different treatments directly affect the TME, new layers of patient-specific response and heterogeneity do not just develop, but are introduced [18]. Individual therapy response due to tissue heterogeneity harshly complicates randomized controlled trials designed with fixed treatment protocols, especially when focused on TME interventions, such as immunotherapeutic approaches [19]. Particularly here, the evaluation of the potency of a fixed treatment protocol (i.e., controlled conditions) might be affected by the patient-specific individual (i.e., uncontrolled) evolutionary dynamics of both the tumor and its TME.

Modern methods, such as DNA, RNA single cell sequencing (sc-seq), methylation profiling and micro-RNA (miRNA) profiling, have made great strides in clarifying tumor and TME development and heterogeneity, making it more possible to pinpoint which patient or tumor characteristics affect response to treatment, and which can be expected to respond. TME immune cell infiltration clusters (GTMEI) have been defined, describing genomic characteristics, molecular subtypes and clinicopathological features as well as proteomic, phosphoproteomic, acetylomic, lipidomic and metabolomic properties [20]. To gain insight into the TME landscape of GBM, Zhao and colleagues assessed expression data of 25 immune cell types from 796 GBM patients. Data could be categorized according to miRNA expression (mi1 to mi5), DNA methylation profiles (dm1 to dm6), IDH status (mutant, wild-type), Gender (female, male), multi-omics (IDH mutant and nmf1 to nmf3), MGMT promoter methylation status (methylated, not methylated), age (less or above 55y), immune profile (im1 to im4), pathologic subtype (classical, IDH mutant, mesenchymal, proneural), WHO grade (high, low) and GTMEI-score: the quantification of immune infiltration patterns in any individual GBM sample [20].

Treatment is standardized for all GBM subtypes and GBM tumor status: despite the fact that tumors can have a different cause and a different developmental trajectory. Similarly to how each person differs from one another, no two tumors are alike. Even within the same person, tumor subclones might develop completely differently depending on its TME. In a RNA sequencing analysis of 107 patients suffering from GBM IDHwt, two subgroups could be identified based on specific gene regulation patterns [21]. These responder subtypes were found to be cancer-cell intrinsic and influenced by the TME. This is only one example of how the heterogeneity of GBM subtypes can impact patient prognosis and warrant variations in treatment. A better understanding of GBM subtype heterogeneity and their developmental trajectory will have a strong impact on course of treatment in a patient-specific way. To this end, new clustering techniques are being developed [22,23]. The rise of personalized therapy makes this realistically possible [24,25,26].

When considering the TME, the relative composition of 24 different types of infiltrating immune cells, together with checkpoint gene expression levels of CD27, PDL1 and CTLA4, can predict GBM prognosis from patient datasets. Three immune subtypes can be distinguished [27]. In immune subtype M1, central memory T cell (Tcm), T follicular helper cell (Tfh) and B cell subpopulations were most enriched. In M2, the top three highest enrichments include Tcm cells, GAMs and Tfh cells. In M3, the top three highest enrichments were Tcm cells, GAMs and B cells. The M3 subtype correlates with the worst prognosis, with high levels of immunosuppression, phagocytosis, leukocyte migration, and TNF superfamily members and -receptors. The M2 subtype, with the best prognosis, exhibited high enrichment scores in CD8+ response, B cell activation, B cell receptor signaling pathway activity, and transcription factor upregulation.

Therapy resistance, heterogeneity, fast development, a complex collection of important tumor characteristics, and predictive immune subtypes based on TME-tumor interactions, all highlight the complexity of GBM, a highly regulated heterogeneous system with spatio-temporal fluctuations that affect treatment response. These characteristics should be considered for individualized treatment plans, which should be adapted according to key GBM tumor and TME changes over time.

Though all fourteen cancer hallmarks play a significant role in the development of GBM, we will outline five key features that not only play an essential role in GBM tumor development, but can be approached with currently available treatment strategies. Listed numerically (1–5) in the order in which they will be discussed in text, each hallmark is also indicated by their icon ID-number: **(1) Cancer stem cells and Glioma stem cells (GSCs) (hallmark 14); (2) Hypoxia (hallmark 5); (3) Metabolic reprogramming (hallmark 7); (4) Immune suppression and inflammation (hallmark 9); and (5) Neuron-glioma interaction (hallmark 1)**. These features are dynamically inter-connected, strongly influenced by the TME, and heterogeneously spatio-temporally represented.

### 2.1. Glioma Stem-like Cells (GSCs)

**Figure 2:**
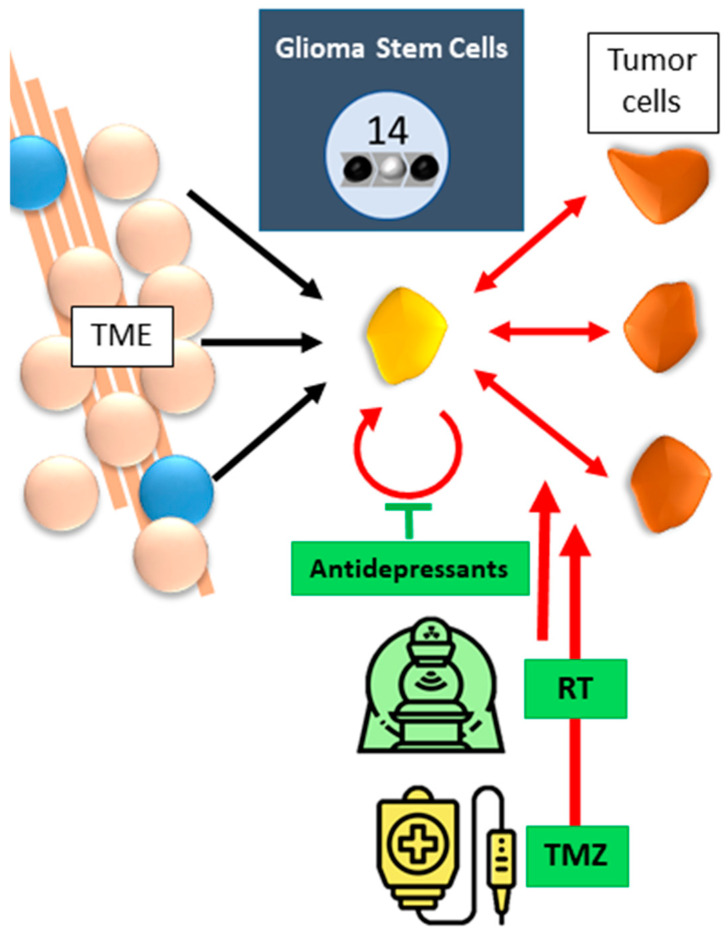
A simplified overview of the effect of glioma stem cells. GSCs can self-renew, differentiate and dedifferentiate to different GBM cell types (red arrows). This can be caused by RT or CTx (TMZ) treatment (red bold arrows). Antidepressants can inhibit GSC plasticity. GSCs rely on a permissive TME (black arrows).

Functional GBM heterogeneity translates to multiple subpopulations of cancer cells [28,29]. Co-operation between genotypically and phenotypically distinct subclones explain the high level of invasion and migration seen in GBM [11]. Based on molecular signature, response to therapy and patient survival, GBM can be subdivided into three categories: proneural, classical and mesenchymal GBM. Comparing the three, the latter is especially linked to a worse patient prognosis. However, recent studies using spatial scRNAseq analysis have indicated that influences from the TME orchestrate a spatial and temporal fluctuation between the three subtypes within one tumor [30,31,32]. Often, all three subtypes can be found, with one subtype highly represented. External influences can cause transdifferentiation, especially towards the mesenchymal GBM subtype [33]. This further complicates efficient treatment. This cellular heterogeneity originates from a small subpopulation of glioma stem-like cells (GSCs, tumor hallmark 14, Figure 2). Less than 1% of tumor cells display GSC-specific properties of self-renewal and multi-lineage differentiation into neurons, astrocytes, and oligodendrocytes. This occurs in three ways: (1) dedifferentiation or reversal of a not yet terminally differentiated to a proliferating form, (2) blocked differentiation, or (3) transdifferentiation, during which cells of a particular phenotype undergo a morphological change, regain their proliferative abilities, and become clearly recognizable as elements of another tissue [3,28]. These three mechanisms underlie the formation of primary tumors, the progression of malignant cell growth, and affect response to therapy. GSCs are inherently resistant to GBM treatment [28,34,35]. They can enter a quiescent status and so escape targeted CTx, and RCT, enabling cancer recurrence. GSCs communicate with their direct TME by cell–cell interaction via tunneling nanotubes (TNTs), causing a TNT-mediated transfer of mitochondria and thereby the modification of the target cell energetic metabolism with increased OXPHOS and ATP production [28]. TNTs differ from tumor microtubes in size (<1 μm width and 30 μm in length to 1.7 μm in width and >500 μm in length, respectively) and life span (TNT: 60 min., TM: >200 days) [36]. The GSC slow metabolism, efficient DNA damage response, and high drug efflux by ABC transporters, further aid GSCs to survive CTx and RCT. Furthermore, RCT and CTx such as Temozolomide (TMZ) consistently increase the GSC pool by causing further genetic mutations and triggering dedifferentiation in non-GSCs. Due to GSC development, GBM recurs more aggressively than the original tumor. It is considered vital to eliminate GSCs for complete tumor eradication. Due to their resistance to SoC treatment, suggested alternatives target GSCs through their deregulated metabolism [28]. Antidepressant drugs such as imipramine, amitriptyline, fluoxetine, mirtazapine, agomelatine, and escitalopram, are prescribed in cancer therapy to combat the side-effects of CTx. However, they were found to silence the phenotypic signature of GSCs, consisting of high expressions of CD44, Ki67, Nestin, Sox1 and Sox2. Particularly imipramine and amitriptyline were reported to modulate GSC plasticity and partially reverse GBM malignancy. These effects were linked to TME conditions and hypoxic fluctuations [37].

GSCs rely on a permissive specific TME niche. This niche consists of many different cell types, from stromal to immune cells, with many reciprocal communications essential for GSCs maintenance, survival and proliferation, as well as TME recruitment, activation programming, and persistence (Figure 2) [35]. Environmental factors originating from the TME affect the bidirectional plasticity of GSCs. For instance, hypoxia promotes a stem-like phenotype in non-GSCs, and the activation of epithelial-to-mesenchymal transition (EMT) further stimulates dedifferentiation of non-GSCs [35].

### 2.2. Hypoxia

Vascularization (tumor hallmark 5) normally occurs through a counterbalance of positive and negative signals, and consists of a complex interplay between soluble factors, membrane bound receptors, integrins and adhesion molecules. Tumors either interfere with regulatory integrins, or disrupt the balance between pro- and anti-angiogenesis signals. Balance disruption can occur through the overexpression of vascularization-stimulating factors, such as vascular endothelial growth factor (VEGF) through oncogene *ras*, or through downregulation of angiogenesis inhibitor factors such as thrombospondin-1 through loss of p53 function [1]. There exist six potential mechanisms of GBM vascularization: (1) Sprouting angiogenesis: the most common way of blood vessel formation via proliferation and migration of endothelial tip cells, (2) Vasculogenesis: Endothelial progenitor cells (EPCs) differentiate into new endothelial cells and form new vascular lumen, (3) Vessel mimicry: tumor cells form vessel-like channels, (4) Vessel Co-option: Tumor cells hijack existing blood vessels, (5) Cancer cell trans-differentiation: cancer stem cells differentiate into endothelial cells and form new vascular lumen, and (6) Intussusception: an existing blood vessel is split into two through mediated cell reorganization [29]. Through these mechanisms, GBM is capable of promoting tumor angiogenesis. However, this dysregulated process, combined with swift tumoral expansion, results in structurally and functionally abnormal vasculature, leading to areas of hypoxia [29,38,39,40]. Hypoxia-related vasculature plays a crucial role in tumor initiation and progression.

Within TME and tumor tissue, two types of hypoxia occur: chronic and cycling hypoxia (also called intermittent hypoxia) [41,42]. Chronic hypoxia is the first type to develop in tumors, and indicates a continuous (≥several hours) state of oxygen deficiency due to limited oxygen diffusion. Cycling hypoxia occurs due to inefficient perfusion in tumor blood vessels [43] and rapid changes in intracellular oxygen requirements due to metabolic changes. It marks fast fluxes between deep (<0.1 mmHg) and moderate hypoxia (15 mmHg). Both chronic and cyclic hypoxia occur with spatio-temporal fluctuation, forcing cells to adapt fast and collaborate for their needs. Cyclic hypoxia translates to a worse prognosis, as it affects many other cancer hallmarks: angiogenesis, intratumoral inflammation, immune evasion, swift cellular adaptation pushing tumor metastasis, intratumoral heterogeneity, and resistance to treatment. In addition to that, the reoxygenation periods give rise to reactive oxygen species (ROS) [41,42].

**Figure 3:**
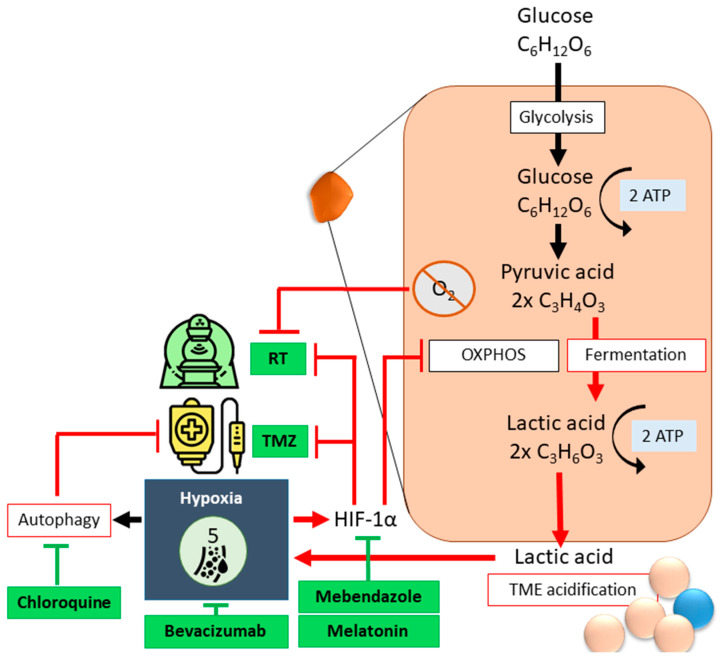
A simplified overview of the effects of hypoxia in GBM. GBM Effects are displayed with red arrows, treatment options are shown in bright green. Hypoxia normally leads to autophagy (black arrow) which inhibits the effects of RT and CTx such as TMZ. Chloroquine treatment can inhibit autophagy. Bevacizumab inhibits angiogenesis factor VEGF. Hypoxia also includes upregulation of HIF-1α, which inhibits both CTx effects and the OXPHOS pathway, driving cellular metabolism toward anaerobic fermentation. Mebendazole and melatonin can be used to normalize HIF-1α expression levels.

Hypoxia leads to SoC-resistance. During RCT, oxygen increases DNA damage through free radical formation; thus, anoxic cells require a radiation dose three times higher to obtain similar effects to treated oxygenated cells [41]. Under hypoxic conditions, metabolic pathways become so dysregulated in GSCs that tumor cells must fall back on autophagy to obtain energy. Autophagy also functions as a protective mechanism against CTx in GBM. Cycling hypoxic tumor cells isolated from GBM xenografts using flow cytometry exhibited higher ABCB1 expression and CTx resistance than chronic hypoxic cells and normoxic cells [41]. Hypoxia-inducible factor (HIF)-1α is a common driver of pathways that define GBM aggressiveness (Figure 3). Downregulation of HIF-1α activity is associated with increased responsiveness to TMZ [38,39,40,41,42,44] and RCT [41,42]. The deregulation of HIF-1α due to hypoxia can be combatted with Mebendazole, an antihelminthic benzimidazole [45]. GBM patients treated with chloroquine, an autophagy flux suppressant, display reduced chemoresistance and improved survival (Figure 3) [46]. According to clinical trials, the nontoxic hormone melatonin is an antiangiogenic, antiproliferative and metastasis-inhibitory agent that enhances the effectivity of other therapeutics when used in combination [47,48]. In GBM, melatonin was found to suppress the HIF-α/VEGF/MMP9 axis through miRNA regulation [49]. As a modulator of the circadian rhythm, melatonin is normally present in the blood and cerebrospinal fluid (CSF). Melatonin concentrations reach a maximum of 80 to 120 pg/mL between 02:00 and 04:00, and drop sharply to between 10 and 20 pg/mL during the day. These inherent temporal fluctuations affect doses and time of administration [48]. Bevacizumab, or Avastin, targets VEGF and specifically combats tumor angiogenesis [50].

It was recently found that some malignant glioma cells can maintain a glycolytic metabolism despite their hypoxic environment by macrophage-mediated recycling of myelin cell debris [51]. Upon scavenging cholesterol-laden myeloid cell debris, GAMs develop into lipid-laden macrophages (LLMs). These LLMs are epigenetically rewired to display immunosuppressive features and are especially enriched in aggressive mesenchymal GBM. Through dynamics in correlation with GBM stage, cellular subtype composition and local tumor niches, LLMs transfer their myelin cell debris cargo to cancer cells, fueling their tumorally increased metabolic demands [51]. This again clearly displays the closely controlled spatial intra-cellular interaction between TME and tumor, and depicts how the development of hypoxia within tumor tissue affects the cellular metabolic programming of both healthy and diseased tissue.

### 2.3. Metabolic Reprogramming

**Figure 4:**
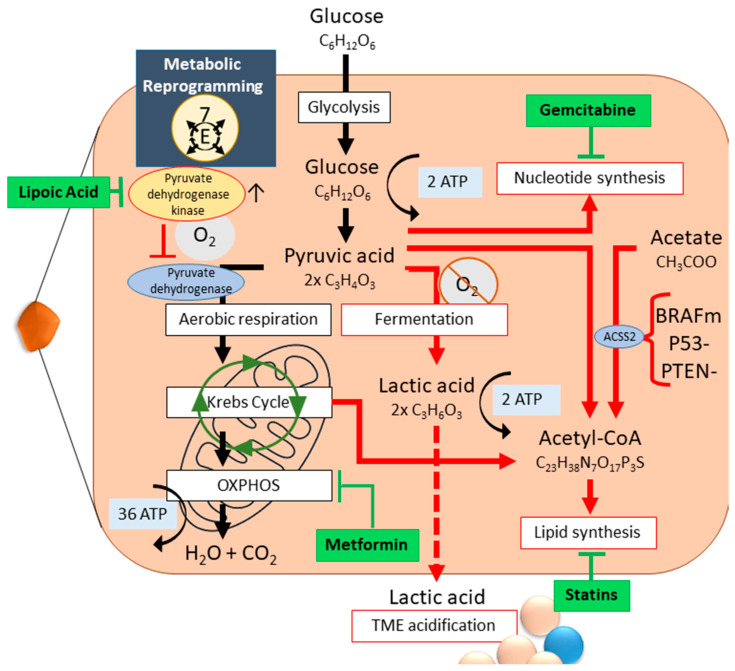
A simplified overview of metabolic reprogramming in GBM. Normal pathway steps are depicted with black arrows, GBM pathway steps are shown in red. Possible treatment options are depicted in bright green. Glucose is processed through glycolysis to intermediates, ATP and NADH. In cancer cells, an increase in pyruvate dehydrogenase kinase (PDK) inhibits aerobic respiration, favoring an anaerobic fermentation pathway instead. Lactic acid production leads to the acidification of the TME. Treatment with lipoic acid inhibits PDK. Metformin can then be used to inhibit the OXPHOS pathway in all fast-growing cells, specifically targeting tumor cells. Combining TMZ with Metformin treatment can revert chemoresistance.

We will zoom in on three different levels of GBM metabolic reprogramming, which strongly affect the aforementioned hallmarks: energy generation, lipid metabolism and nucleotide metabolism (Figure 4) [52].

Under normoxic conditions, healthy cells process glucose through glycolysis and aerobic respiration to pyruvate and subsequently to CO_2_, generating 38 ATP. In tumor cells, including GBM, the glucose, glutamine and fatty acid metabolism are heavily altered [34] (Hallmark 7, Figure 4). Due to increased proliferation, tumor cells opt for aerobic glycolysis, known as the Warburg effect [53,54]. The intermediary glycolysis products are used for cellular growth, inhibiting aerobic respiration [34]. Lack of oxygen and redirection of necessary intermediary products pushes anaerobic fermentation, generating only four ATP [34]. Metabolic reprogramming leads to the development of different intratumoral subpopulations which collaborate to ensure an all-round sufficient nutrient supply, ROS clean-up, and energy (Figure 4) [39,40]. As a result of metabolic reprogramming and hypoxia, the electron transport chain collapses, leaving the cell with acute ATP depletion. Areas of necrosis develop within the tumor [55]. Even without hypoxia, necroptotic areas occur. Necrosis in GBM is a well-regulated malignant process: it involves neutrophil-triggered ferroptosis; accumulation of neutrophils within GBM correlates both temporally and spatially with necrotic areas. Neutrophil depletion dampens necrotic area occurrence [56]. GBM necrosis induces massive angiogenesis through VEGF expression and releases high mobility growth factor (HMGB-1), promoting tumor proliferation and invasion by elevated IL-8 expression in peri-necrotic regions [55,57]. Cytosolic proteins released during necrosis elicit a pro-inflammatory cytokine response, which aid in angiogenesis, GBM migration, and chemo-resistance [55]. Necrotic foci in GBM are considered a pathological and radiologic hallmark, signaling an overall poor prognosis. GBM patients whose cells do employ the OXPHOS pathway have a better prognosis [52,58]. OXPHOS exclusion is enriched in the mesenchymal GBM subtype, and is, as noted before, linked to SoC resistance.

Lipids are fat-soluble organic compounds with structural, signaling and storage functions. The TME of GBM is a lipid-rich environment, as the brain dry-weight naturally exists for 50% of lipids. The side-products of glycolysis and the Krebs cycle, normally used for energy generation, are redirected for cytosolic acetyl-CoA synthesis. Simultaneously, extracellular acetate import is upregulated and used to produce acetyl-CoA via acetyl-CoA synthase (ACSS2), which is upregulated through BRAF, p53 and PTEN mutations and malfunction, common in GBM [52]. GBM requires elevated levels of de novo lipogenesis for proliferation, and not only as a structural component: GSCs rely heavily on fatty acid oxidation. Thus, the aberrant lipid metabolism is heterogeneous throughout tumors, and combination therapies are advised. The lipid metabolism, including the fatty acid oxidation and de novo lipogenesis, is a potential target for statins (Figure 4) [52].

As important signaling and structural components, nucleotide production is highly upregulated in GBM. Nucleotides can be synthesized de novo or via salvage pathways: GBM relies heavily on the former, which is orchestrated by c-MYC activity. However, salvage pathways are also used. The ability of GBM cells to scavenge hypoxanthine, the most abundant purine present in the CSF, is thought to cause GBM anti-folate therapy resistance [52]. Gemcitabine is a cytidine analog which, when incorporated into the DNA, inhibits ribonucleotide reductase. Gemcitabine can cross the BBB and accumulates in GBM [59].

As the source of nutrients and oxygen, the TME plays a major role in GBM metabolic reprogramming. For instance, hypoxia activates aerobic glycolysis and triggers EMT through HIF. Metabolic reprogramming and release of ROS accelerate TME acidification. GSCs are dependent on strong metabolic reprogramming, mitigated by the TME [33,35]. Metformin combats cancer cells in three distinct ways, effectively targeting both non-GSCs and GSCs through their aberrant metabolism [35,60]. Firstly, metformin rewires the metabolic function from anaerobic fermentation back to glycolytic. Transcriptional analysis of macrophages derived from metformin-treated mouse bone marrow cells show a decreased expression of pro-tumor genes such as transforming growth factor (TGF)-β and interleukin (IL)-1β [60]. Secondly, metformin targets tumor cells that use glycolysis by blocking glucose uptake and inhibiting complex I of the mitochondrial electron transport chain to disrupt oxidative phosphorylation (OXPHOS) and subsequent mitochondrial ATP generation. The lower glycaemia, lower glucose uptake, and lower ATP levels in tumor cells, also due to hypoxia, inhibit mTOR complex 1, a major hub for cell growth, protein translation and cellular metabolism. Thirdly, metformin plus TMZ can revert chemoresistance under hypoxic conditions via PI3K/mTOR pathway suppression (Figure 4) [61].

### 2.4. Immune Suppression and Inflammation

**Figure 5:**
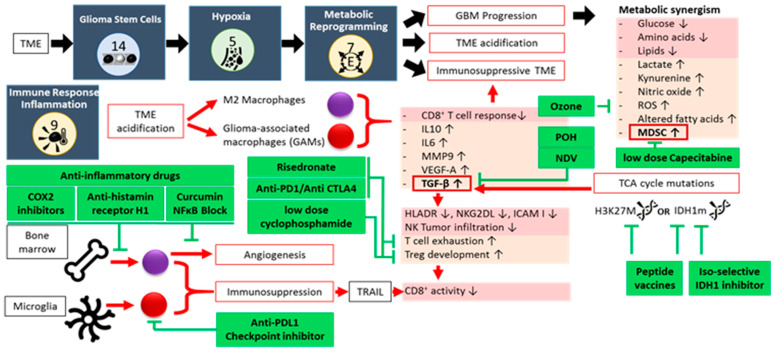
A simplified overview of the effects of immune response inflammation in GBM. Tumor-specific occurrences are indicated with red arrows, possible treatment options and effects in bright green. Downregulation (downward black arrows) of cell types or cytokines is highlighted in red, and upregulation (upward black arrows) is highlighted in orange. Due to TME acidification, M2 Macrophages and GAMs are upregulated, which inhibits cytotoxic T cell response but increases inflammation-regulating cytokines, developing into an immunosuppressive TME. Especially the upregulation of TGF-β, by both tumor-associated macrophages as well as TCA cycle mutations, further inhibits the innate and adaptive immune system. TCA cycle mutations can be halted with ONC201 treatment, isoselective inhibitors and peptide vaccines. GAMs, especially when derived from bone marrow, also aid angiogenesis. PDL1+ M2 Macrophages target CD8+ T cells via the apoptotic TRAIL pathway, both during CD8+ T cell priming and effector function. Using anti-PDL1 checkpoint inhibitors, this PD1-independent immunosuppression can be inhibited.

Cell-to-cell TME and GBM interactions, metabolic alterations, spatio-temporal oxygen fluctuations, and cellular necrosis together create a constant state of inflammation within the TME (hallmark 9, Figure 5). Rather than help combat the tumor, this perpetual inflammatory environment enhances tumorigenesis and progression by promoting other cancer hallmarks. Pro-inflammatory biomolecules drawn to the inflamed TME include: (1) growth factors that sustain proliferative signaling; (2) survival factors that inhibit apoptosis; (3) proangiogenic factors that nurture vascularization, which in turn promotes invasion and metastasis; and (4) inductive signals that lead to EMT activation. Multiple occurrences are known in which a perpetuating inflammation instigated tumor formation. A vicious circle develops, resulting in an immunosuppressive TME [41], supporting critical immune components such as MDSCs and GAMs [58,62,63,64,65]. The alteration of glycans and lectins further promotes tumor progression and immune evasion by enhancing immunosuppressive cell subsets while impairing immune effector populations. These pathways, along with their associated receptors (C-type lectins, Siglecs, and galectins), represent promising therapeutic targets [66].

The interplay between GBM and MDSCs results in a metabolic synergism, which causes further deprivation of glucose, amino acids and lipids, as well as accumulation of lactate, kynurenine, nitric oxide (NO), ROS, and altered fatty acids. This stimulates further MDSC recruitment and activation, immunosuppression, reduction in T cell responsiveness, and TME acidification [67]. Under acidic conditions, macrophages are polarized to the M2 phenotype [68]. A recently discovered immune suppression mechanism involves PERK-driven glucose metabolism in M2 macrophages. Through glycolysis-dependent histone lactylation, a recently identified post-translational modification, a IL-10 expression and a T-cell suppression are promoted in GBM [69]. Lactylation regulates gene transcription and is closely linked to the Warburg effect, with lactate as the key driver in the TME. This modification occurs on histone and non-histone proteins, including oncoproteins, and is implicated in various physiological and pathological processes [70]. The continuous low-dose, or metronomic, administration of capecitabine, an orally available 5-fluorouracil (FU) CTx prodrug, reduces circulating MDSCs and increases cytotoxic immune cell tumor infiltration [71]. Ozone therapy can further reduce ROS [72].

Microglia/macrophage functions are almost immediately altered upon exposure to the GBM TME secretome, and give rise to GAMs [64], which make up about 50% of total live GBM biomass [73]. GAMs in GBM further contribute to the construction of the immunosuppressive TME by expressing high levels of IL-10, IL-6, TGF-β, angiogenic molecules (VEGF-A), matrix metalloproteinases (MMP9), and by inhibiting CD8+T cell infiltration [74,75]. GAMs originate from two independent sources: bone marrow (85% in GBM) and microglia (15% in GBM). Each subtype performs different functions at different stages of tumor progression [18,76,77]. Though macrophages and microglia in GBM have morphological and behavioral changes, it was deemed problematic to distinguish between the two. Advanced methods including scRNAseq, cellular indexing of transcriptomes and epitomes by sequencing (CITE-seq), and cytometry by time of flight (CyTOF) have shown that macrophages show enriched levels of CCR2, CD45RA, CD141, ICAM, CD1C, CD1B, TGFBI, FXYD5, FCGR2B, CLEC12A, CLEC10A, CD207, CD49D, and CD209, whereas microglia express high levels of CX3CR1, SALL1, HEXB, P2RY12, and TMEM119 [73,78]. Primary GBM is more likely to be infiltrated by GAMs derived from microglia, whereas they are absent from the core of metastatic brain tumors, which are mainly inhabited by bone marrow-derived GAMs [73]. The polarization of microglia or bone marrow-derived macrophages has been suggested to mediate the immunostimulatory effects of certain treatments, including RT. The more dynamic M2 macrophages increase tumor growth and suppress immune responses, whereas the more-defined M1 subtypes reduce tumor growth, improving RT efficacy [18]. The bone marrow-derived macrophage population exerts immunosuppressive and proangiogenic effects. Anti-inflammatory drugs like COX2 inhibition [79], anti-histamine receptor H1 drugs [80] and curcumin [78] can influence their biological role. Microglia are unique to tumors in the CNS. These main resident myeloid cells play a crucial role in the surveillance, development and maintenance of the brain [64,65,73]. They are involved in neurogenesis and axonal growth, and orchestrate immune responses against pathogens or damaged cells. Microglia exhibit a broad functional diversity and can be classified into multiple subtypes based on their characteristics, which depend on the brain developmental stage and tissue location [81,82]. They switch between different functional states depending on microenvironmental cues. (Figure 5).

Due to their aberrant expression patterns, GAMs can be targeted for treatment, though thus far with limited success [73]. GAMs overexpress PDL1, TNF and TNF-related apoptosis inducing ligand (TRAIL), but not Fas ligand [83]. Of particular interest is the finding that PDL1-expressing GAMs block CD8+ T cell responses (priming and effector function) via TRAIL signaling [83]. This suggests that targeting GAMs via PDL1 can rescue CD8 T cell responses upon dendritic cell vaccinations.

The resident T cell population in the TME is heavily altered by tumor development. Cytotoxic activity is downregulated by promoting T cell exhaustion, and by an increased presence of Tregs. Checkpoint inhibitors targeting T cell surface markers, such as Anti-PD1 or Anti-CTLA4, inhibit cytotoxic T cell exhaustion by tumor cells [84]. Nitrogen-containing bisphosphonates, such as risedronate, have a direct anti-proliferative effect on tumor cells through Ras and Rab modification [85], but also reactivates cytotoxic T cell activity [86]. Monoterpene and Ras inhibitor perillyl alcohol (POH) have been shown to significantly increase overall survival in GBM patients, independent of their MGMT status, by inhibiting the cell cycle. They also act through TGF-β, the endoplasmic reticulum (ER) stress pathway, and by arresting survival pathways such as mTOR. When administered intranasally, POH crosses the BBB and thus affects GBM tumors which are otherwise difficult to reach [87,88]. Cyclophosphamide (CPM) is an alkylating CTx agent. Similarly to risedronate, CPM has direct cytotoxic and immune-modulatory effects. Low dose CPM has been shown to deplete regulatory T cells [89] and increase CD4+ and CD8+ T cells in the TME, as CPM downregulates TGF-β receptor 2 expression levels [90,91]. It was found most effective as a metronomic administration, resulting in a reduced myofibroblast population [91,92].

A critical link between the aberrant glycolysis and immunosuppression is the production of TGF-β [63]. TGF-β down-regulates tumor surface antigens such as HLA-DR and NKG2DL, and intercellular adhesion molecule I. Infiltration of NK cells in early stage of tumorigenesis is inhibited by TGF-β. TGF-β plays a role in T cell exhaustion. Through TGF-β signaling, CD4+CD25− naïve T cells develop into T-regs that inhibit tumor-specific CD8+ T cell cytotoxicity and NK cell function. However, aberrant TGF-β production also weakens the tumor cell immune system, allowing oncolytic viruses otherwise unable to infect human cells, such as Newcastle Disease Virus (NDV), to specifically infect and kill the tumor from the inside out [93,94]. Immune function is further hampered by mutations in TCA cycle-enzymes. The accumulation of the oncometabolite D-2-hydroxyglutarate in IDH-mutated gliomas contributes to the “cold” tumor immune phenotype [95]. The mutation of IDH1 suppresses STAT1, a regulator of CXCL10, leading to reduced CD8+ T cell accumulation in gliomas [96]. The “cold” tumor immune phenotype might shape the TME, allowing the development of tumor subclones with higher-grade features. Isoform-selective IDH inhibitors and peptide vaccines targeting IDH-mutant gliomas have shown translational potential in preclinical and clinical studies by interfering with both the cellular metabolism and the relative composition of target-specific tumor-infiltrating cytotoxic T cells [97,98]. Overall, the aberrant metabolism creates metabolic checkpoints underlying the immunosuppressive TME [99].

### 2.5. Neuron-Glioma Interaction

Neoplastic cells elicit sustained proliferative signaling (hallmark 1) in three ways: (i) autocrine signaling; (ii) overexpression or structural alteration of membrane-bound growth signal transducers; and (iii) disruption of intracellular matrix receptors (integrins), favoring transmission of proliferative signals [100]. The latter occurs in at least 30% of tumors, including melanoma, breast cancer, ovarian cancer, colon cancer, thyroid cancer, prostate cancer, and glioblastoma [100,101].

**Figure 6:**
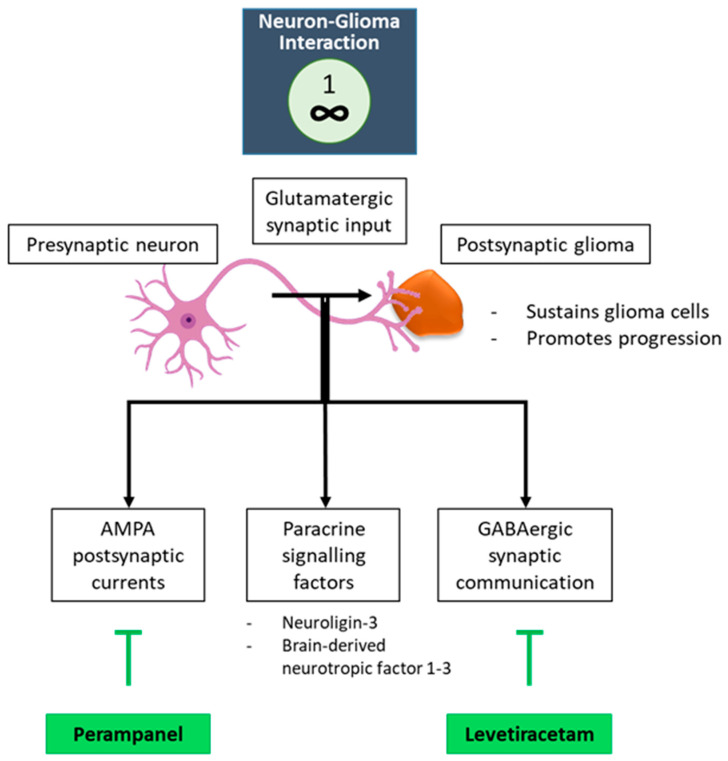
A simplified overview of the effects of the neuron-glioma interactions. The interactions between presynaptic neurons and postsynaptic gliomas drive tumor development through AMPA postsynaptic currents, paracrine signaling factors, or GABAergic synaptic communication. The first can be inhibited with perampanel and the last one with levetiracetam.

Not considering certain TME and tumor-specific aspects, the key features discussed up to now play a role in any type of tumor. However, there is one key feature truly unique to GBM that bears consideration: the neuron-glioma axis (Figure 6) [15,16,73,102,103,104,105]. Neuronal activity drives tumor progression through paracrine signaling factors such as neuroligin-3 and brain-derived neurotrophic factor [1,2,3]. A direct glutamatergic synaptic input from presynaptic neurons to postsynaptic glioma cells sustains the glioma cell network and drives glioma progression [16,105]. Postsynaptic currents are mediated by glutamate receptors of the AMPA subtype, which can be targeted with the AMPA receptor antagonist perampanel (Figure [16]. Levetiracetam can target the GABAergic synaptic communication node [106]. Direct neuro-immune interactions occur via GABA-associated immune cell subtypes like M2 macrophages and T cells, contributing to tumor immunosuppressive mechanisms (Figure 6) [107]. Knowledge in the domain of cancer neuroscience is growing fast, as several cancer hallmarks intersect with neuroscience in the CNS TME [108].

## 3. GBM Versus Pediatric-Type Diffuse High-Grade Gliomas

Brain tumors are the leading cause of cancer-related child mortality [109]. Diffuse midline gliomas (DMG) are considered one of the most aggressive pediatric brain malignancies. DMGs, also known as diffuse intrinsic pontine glioma (DIPG) when located in the pons, typically affect children between the age of 5 and 10, but also occur in adolescents and adults. DIPG is a uniformly fatal diagnosis, with a median survival of less than one year, due to its location in the brainstem which makes resection impossible. Over a hundred clinical trials and testing methods such as radiosensitizing agents, cytotoxic or high-dose chemotherapy with or without stem cell rescue, or molecularly targeted agents, showed no OS improvement over RT. Even if a child makes it through harsh rounds of treatment, the damage done to the developing brain results in much more severe loss of quality of life than adults who undergo the same SoC. Often, pediatric treatment is thus focused on minimizing toxicity [110]. Due to the many challenges in studying pediatric brain malignancies, progress in understanding tumor development and immunotherapy is slow [81]. Many pediatric brain tumors are thought to arise during prenatal brain development as embryonal tumors, and coincide with periods where unique subpopulations of immature microglia are abundant, which are therefore implicated in pediatric malignancies [81,110]. Although some childhood brain tumors share similarities with adult GBM, many are distinct entities, residing in locations different from adult brain tumors, and thus have a completely different TME. Mechanistic characteristics and treatment methods can be compared across adult-type diffuse glioma, particularly IDH-wildtype GBM, and diffuse pediatric high-grade gliomas. We will specifically elaborate on diffuse pediatric-type high-grade glioma (pDHGG) H3 wild-type and IDH1 wild-type, diffuse hemispheric glioma H3G34-mutant (H3 G34-mutant DHG), and diffuse midline glioma H3K27-altered (H3 K27-altered DMG) [4,111].

Similarly to adult GBM, pediatric-type diffuse high-grade gliomas are highly heterogeneous [112]. The immune and inflammatory cellular players in the TME are quite similar to the adult counterpart; the major immune infiltrates are macrophages and T cells, though they are extremely sparsely available [77,113]. It was suggested that as the tumor develops from low to high grade malignancy, a transient change in the relative composition of T cells and myeloid cells occurs: effector T cells make room for Tregs, microglial cells diminish, GAMs increase and give rise to a large population of monocyte-derived M2 macrophages. The TME in pediatric-type diffuse high-grade gliomas is characterized by hypoxic regions and low nutrients, similar to the GBM TME, which translates to an immunosuppressive environment. However, there exist significant general and specific differences between pediatric and adult malignant gliomas, both on the genetic and epigenetic level [77,81,113]. As the CNS develops, a diversity of unique immature microglia is abundantly present, which presumably not only contributes to tumor onset and progression, but results in a glioma characterized by a relatively high number of microglia with aberrant morphologies and phenotypes. This suggests a crucial role in TME development [77,81]. Complications and treatment options connected to glioma-neuron interactions therefore carry more weight in pediatric-type diffuse high-grade gliomas, in comparison to adult GBM. Pediatric tumors need more frequent monitoring, both to balance out the faster change in a developing brain compared to a fully developed, adult brain, but also as this “young” environment is more prone to GSC occurrence. Children with brain tumors are thus more prone to develop radiotherapy resistance, and are simultaneously more vulnerable to RTs neurotoxic effects [110,114]. Immunotherapeutics are considered an answer to this toxicity problem: their ability to target the tumor specifically minimizes organ damage and allows for tumor treatment despite its inaccessible location. This, however, comes with complications: adult clinical trial results cannot be used for pediatric patients, as their immune system is not yet fully developed. Compared to adult GBM, pediatric malignancies have a low mutational burden, which means checkpoint blockade strategies are less effective. Adding to this is the fact that immunotherapy development has often been carried out through research on adult GBM in immunocompromised mice. Only scant evidence exists on immunotherapies in children, due to ethical objections and fast disease progression [110].

The diffuse pediatric-type high-grade glioma (pDHGG) (H3-wildtype and IDH-wildtype), historically known as pediatric GBM, shares a similar immune response to adult GBM based on its immune infiltration profile [81]. Both have an increased anti-inflammatory myeloid cell population with high M2 and PD1 marker expression and a reduced CD8+ T cell and NK activation rate. Both contain necroptotic areas. Spatially resolved single-cell analysis of diffuse pDHGG (H3-wildtype and IDH-wildtype) have indicated adenosine-rich regions harboring specific immunomodulatory functions involving microglia. The spatial distribution of immune cells within the TME is considered crucial for understanding and identifying targetable vulnerabilities in pDHGG (H3-wildtype and IDH-wildtype) [81].

The immune cell composition is different in H3 G34-mutant DHG. Most tumors belong to a pediatric high-grade glioma immune cluster type 3, with moderate levels of CD8+ T cells and relatively low levels of other infiltrating immune cell types [113]. Curiously, a higher than median concentration of CD4+ T cells and NK cells was associated with poor survival. Another study characterized H3 G34-mutant DHG as a tumor with a higher level of immune cell infiltration, with higher levels of monocytes and CD4+ T cells, but lower Treg infiltration [77].

**Figure 7:**
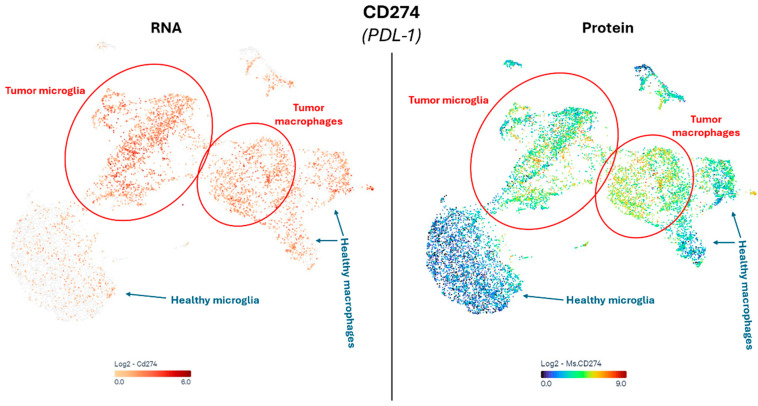
Single-cell RNA and protein analysis of myeloid cells in the pontine region of syngeneic allograft mouse models for DMG, indicating PDL1 expression in GAMs compared to healthy cells. Color scales indicate expression levels. Red circles indicate the different tumor–associated grouped cells.

Mutations of IDH1 and H3 K27M are mutually exclusive, resulting in a completely different phenotype in H3 K27-altered DMGs compared to their IDH1-mutant counterparts, but also when compared to pDHGG (H3-wildtype and IDH-wildtype) and adult GBM [77,115,116]. DMG tumors are generally found to have a comparatively high inflammatory, “cold” TME characterized by a low T cell infiltration. DMG is typically known for a high infiltration of macrophages or microglia, but limited to no T or NK cells, and an absence of chemokine or cytokine expression to recruit these lymphocytes. This does not mean the present microglia are completely inactive: DMG cells can induce a loss of histone 3 lysine 27 trimethylation in microglia and consequently affect its phenotype to a tumor-promoting state, contributing to the highly immunosuppressive conditions within DMGs [81]. However, it was shown that DMG had a greater inflammatory TME compared to the hemispheric high-grade gliomas, including hemispheric pDHGG, and displayed a greater expression of immune-related genes indicating the presence of T cells, macrophages, neutrophils and CD45^+^ cells [117]. The understanding of the biological function of inflammatory cells in DMG progressed fast with the development of highly innovative syngeneic allograft mouse models, of which the histopathology, the immune microenvironment and the therapeutic response of DMG adequately recapitulates human DMG [118]. Of particular interest is the high PDL1 expression in tumor-associated versus healthy microglia and macrophages, demonstrated at both the single-cell RNA and protein level using CITEseq analysis of syngeneic DMG tissues (Figure 7). As previously noted, PDL1 overexpression in GAMs as compared to non-tumor-associated macrophages is prevalent in DMG (Figure 7). GAMs driving T cells from a progenitor exhaustion state to terminal exhaustion has also been described [119].

These data are confirmed by findings in human DMG tissue (communication of unpublished data by DSM and EH). A recent review assessed MDSCs as a potential immunotherapy target in DMG [120]. Overall, these data form the rationale for targeting the myeloid component in DMG treatment. Metabolic alterations in H3 K27-altered DMG cells include an increased glucose dependence for energy with subsequent elevated lactate production levels, an increased cholesterol biosynthesis, increased TCA and OXPHOS metabolism, increased glutaminolysis and increased Pentose Phosphate Pathway activity [121]. An observed increase in DMG citrate concentration (also seen during hypoxia) combined with the reduced relative tumor blood volume detected by advanced magnetic resonance spectroscopy (MRS) and -imaging (MRI), and their improvement upon treatment, strongly suggest that intratumoral hypoxia also plays a prominent role in DMG [122]. GABAergic synaptic communication between GABAergic interneurons and DMG cells were discovered, underscoring tumor subtype-specific mechanisms of brain cancer neurophysiology [106], and suggesting the potential need for the additional blockage of the GABAergic pathway with levetiracetam besides blocking the glutamatergic pathway with perampanel in DIPG [123]. One particular type of dopamine antagonist drug, the first in line being ONC201, emerged recently as promising against DIPG/DMG. ONC201, or dordaviprone, selectively binds dopamine receptor D2 (DRD2) and so inhibits cell proliferation via the Ras pathway [124]. It also binds to mitochondrial caseinolytic protease P (ClpP) which impairs mitochondrial activity and subsequent apoptosis [125]. Though ONC201 treatment does not prolong overall survival of malignant glioma in general, ONC201 in H3 K27-altered DMG does have a positive effect. H3 K27-altered cells use α-ketoglutaric acid (α-KG) to maintain a low methylation status (H3 K27me3). The application of ONC201 increases H3 K27me3 by increasing glutamine over glucose derivation of α-KG, which is then metabolized to 2-hydroxyglutarat instead [124]. As ONC201 activates the ATF4/CHOP-mediated integrated stress response via downstream target engagement, it indirectly activates the TRAIL/Death receptor 5, inactivates Akt/ERK signaling in tumor cells, and inhibits OXPHOS via c-myc [126]. TRAIL-independent apoptosis, cell cycle arrest, and antiproliferative effects also occur. As this extends the effects of ONC201 beyond tumor bulk to include cancer stem cells, associated fibroblasts and TME immune cells, it is unsurprising that clinical studies have shown that ONC201 does not only have a clinical activity on its own but also synergizes with RT, CTx, targeted therapy and immune checkpoint agents [126].

## 4. Need for Innovative Multiphase Individualized Combination Treatment

Though each key feature can be linked to a specific tumor hallmark, all of them are in fact the result of a complex interplay between multiple tumor hallmarks, and closely interact and affect one another. GSCs communicate with their direct TME by cell–cell interaction via TNT, directly affecting the energetic metabolism of their surrounding cells [28]. The oncogenes, which cause malignancy, often directly deregulate the cellular metabolism: TP53 regulates the lipid metabolism in cancer cells, and c-MYC activates glutamine uptake [34]. Glutamine competition halts immune cell activation, and inhibits CD4+ T cell development into inflammatory subtypes. Intermediary and end products of glycolysis and lactate fermentation inhibit NK cell function, Th1 differentiation, B cell function, and T cell activation. Competitive glucose uptake is the main cause of impaired T cell function in the TME [127]. The regulation of vasculature and angiogenesis results in the formation of fluctuating areas of hypoxia and varying nutrient availability. This, in turn, contributes to the deregulation of metabolism, and non-mutational epigenetic reprogramming, promotes tumor tissue heterogeneity, and increases invasiveness and metastasis [41,75]. Oxygen concentrations play a fundamental role in stemness maintenance, defining several GSC niches. Hypoxia even enlarges the GSC population [41]. One can distinguish five hypoxia-related niches [29]: (1) the perivascular niche where proneural GSCs mainly reside; (2) the immune niche; (3) the hypoxia/necrotic niche where mesenchymal GSCs mainly reside; (4) the extracellular matrix niche; and (5) the peri-arteriolar niche. All of these niches also interact with each other.

**Figure 8:**
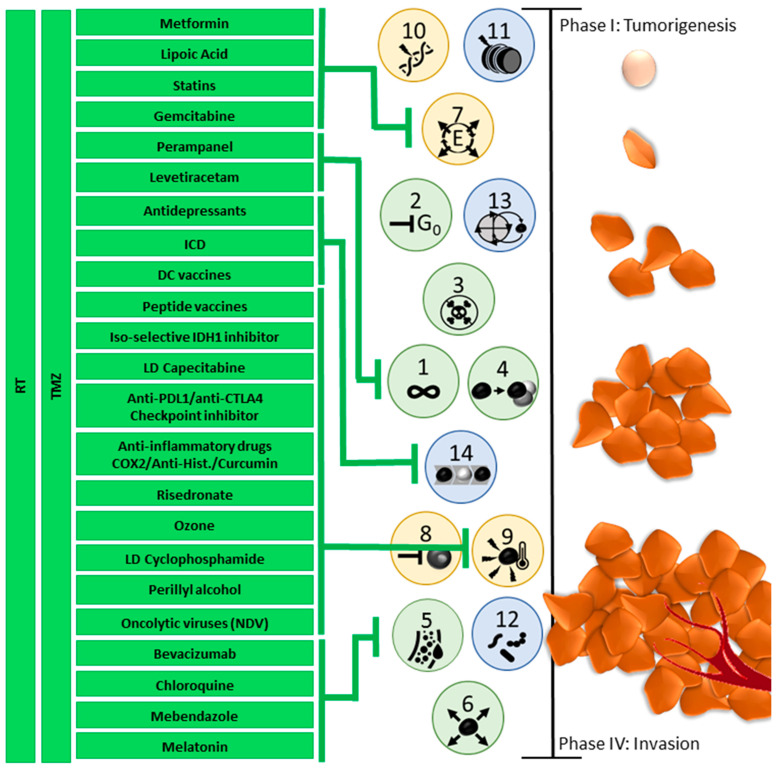
Example of temporal occurrence of individual tumor hallmarks during tumor development, from stage I, Tumorigenesis to stage IV, Invasion. Included is a temporal tumor treatment plan, to combat specific tumor hallmarks throughout malignancy development.

Taken together, these subsequent changes and intratumoral fluctuations create a temporal effect within hallmark development. This explains the heterogeneity seen in tumors, as well as their fast-adapting character over time. An example of this is the fact that when fluctuating areas of hypoxia develop due to angiogenic deregulation and changes in metabolism, different cell populations develop within one tumor which collaborates to regulate nutrient availability throughout the tumor tissue [34]. It also explains why tumors can exhibit such different and fast-changing behaviors and differences in development between people, tumor locations, and over time [3,34,40,75]. Figure 8 outlines how, eventually, the different tumor hallmarks together can influence tumor development, and how repurposed drugs can target different hallmarks. A chance interplay between genomic mutations (hallmark **10**) and non-mutational epigenetic reprogramming (hallmark **11**), deregulates the cellular metabolism (hallmark **7**), which in turn causes a cell to evade growth suppression (hallmark **2**) and leads to subsequent cellular senescence (hallmark **13**) as the cell proliferates. The genomic instability and fast cell division of the starting malignant cells lead to resistance to apoptosis (hallmark **3**). The cells instead gain sustained proliferative signaling (hallmark **1**) and maybe even replicative immortality (hallmark **4**). Phenotypic plasticity is unlocked (hallmark **14**), which not only allows the growing malignancy to avoid immune destruction (hallmark **8**) but helps it evade and affect the immune cells, resulting in a tumor-promoting inflammation (hallmark **9**). The subsequent metabolic changes alter cellular nutrient needs, while the growing tumor develops hypoxic areas, causing the cancer cells to induce vascularization (hallmark **5**) which affects the influx of possible polymorphic bacteria (hallmark **12**). Finally, tumor cells gain the ability to invade healthy tissue (hallmark **6**), causing the tumor to spread throughout the body.

It should be strongly emphasized that this is only one possible scenario of tumor development. Not all hallmarks occur in every tumor cell, not all hallmarks occur simultaneously, the order of hallmark occurrence can vary, and each hallmark can push the cascade of tumor development as a whole. As a source of nutrients, blood vessel transport, microbes, different healthy cell types, and immune cells, the TME plays a major role in how fast the different tumor cells, and thus the tumor entity, have a chance to develop.

**Figure 9:**
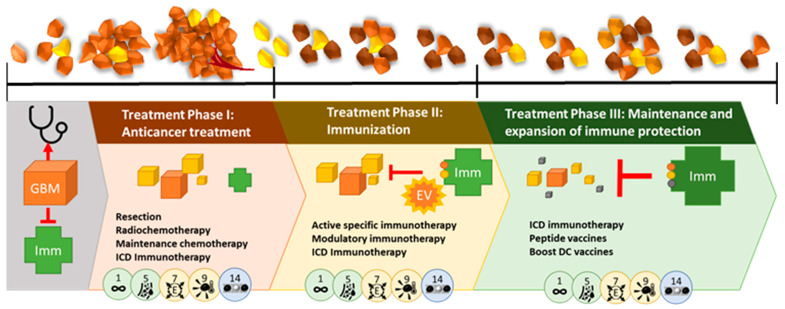
Overview of possible combination strategy trajectory, separated into three treatment phases that allow for adaptation and optimal patient-individualized treatment, based on tumor status and activity. GBM leads to symptoms (indicated as a stethoscope) and suppresses the immune system. Three subsequent treatment phases are proposed to combat the initial tumor and its escape mechanisms, by temporal targeting both, the tumor directly, and its TME. The gray–red–yellow–green background colors reflect treatment effectivity as increased tumor control, also shown as tumor dedifferentiation and size decrease at the top of the figure. Yellow tumor cells represent glioma stem cells, tumor cells in variations of brown (light to dark) represent tumor cells of a different subtype, indicating here how a tumor of a different variation might grow after initial treatment of the main tumor subtype present, and how treatment might push further tumor differentiation.

Based on this knowledge, a temporal treatment plan should be developed, and adapted specifically to each patient (Figure 9). This described treatment strategy is only one example for setting up a hypothetic rational strategy that acknowledges both the evolutionary dynamics of the tumor cells and the TME. Having neurosurgery/radiochemotherapy/alkylants as the only SoC approach, multiple combination treatment approaches can and should enter clinical research. The following described treatment strategy has been pioneered [26,128,129].

Globally, three different phases of combination therapies emerge, each including therapies focused against cancer cells, focused on the cancer-immune interaction and focused on the TME. The different phases ensure an effective response to an adaptable malignant glioma, a heterogeneous TME, and a changing systemic immune compartment. It allows for necessary timely adaptations, depending on current tumor status, and should thus be paired with continuous tumor monitoring.

The first anticancer treatment phase (treatment phase I) aims to reduce tumor size, proliferation and infiltration via SoC treatment (surgery, RCT, and maintenance CTx) with 5 days of TMZ in 4-week cycles or Lomustin/TMZ in 6-week cycles. About 2 days after the maintenance chemotherapy course, immunogenic cell death (ICD) immunotherapy consisting of 5 daily sessions of modulated electrohyperthermia (mEHT) and bolus injections of oncolytic Newcastle Disease Virus (NDV) are given. ICD immunotherapy aims to kill cancer cells, including GSCs, via a biological and physics approach instead of only alkylating agents. As a result, the tumor burden reduces in size. The mEHT functions to prime the immune system in the TME and the inclusion of NDV ensures a cytotoxic immune response. Tumor Treating Fields (TTF), another type of physics therapy based on electromagnetic waves, has already been added at this stage, resulting in improved prognosis [130]. Of note, part of the working mechanism of TTF has been suggested to be ICD-mediated tumor cell killing [131]. During this first anticancer phase, the use of repurposed drugs to affect the TME is proposed: anti-inflammatory drugs (Cox2 inhibition, NFkB inhibition, anti Histamin R1 drugs), a metabolic cocktail (Metformin, Lipoic acid, Atorvastatin), Mebendazole, drugs targeting the neuron-glioma axis, and melatonin. During treatment phase I, the administered RCT and CTx target immune cells besides the tumor cells, thereby weakening the immune system. In addition, the heterogeneous tumor is prone to mutate in response to treatment, with the formation of new subclones with a higher mutational burden. The treatment-induced combination of more aggressive tumor subclones with weakened immune surveillance is life-threatening. By adding ICD immunotherapy during the first anticancer treatment phase, the TME is prepared for a second phase of global treatment: immunization.

Treatment phase II consists of active specific immunotherapy and modulatory immunotherapy to strengthen the immune system and train it against the heterogeneous residual tumor subclones and the GSC tumor cells. Patient-derived dendritic cells (DCs) are loaded with patient-specific tumor antigens. Either or both lysate from the original tumor tissue and ICD immunotherapy-induced serum-derived antigenic extracellular microvesicles and apoptotic bodies, also called large oncosomes [132], can be used to load the DCs. Both contain the spectrum of antigens that are present at time of vaccination, at the end of the first anticancer phase, during which radio- and chemotherapy might have altered tumor subclones and antigenicity. A cytokine cocktail (TNF-α, IL-1β, IL-6) and the addition of NDV, which delivers viral antigen presentation and TLR stimulation, guarantee full DC maturation at time of DC vaccine release. After intradermal injection, the mature DCs reactivate endogenous T cells to target both the tumor itself and the NDV present inside the tumor after phase I. Concurrent modulatory immunotherapy consists of a patient-specific tailored combination of anti-inflammatory agents, checkpoint inhibitors (targeting, e.g., PD1-PDL1 interaction or CTLA4-CD80/CD86 interaction), and bisphosphonates (e.g., risedronate). The use of anti-PDL1 as a checkpoint inhibitor during this immunization phase might be favorable in the treatment of GBM, because of the particular targeting of the GAMs [83]. TME treatment with repurposed drugs is maintained during this phase. The scheduling of DC vaccination after maintenance CTx but not during CTx has been suggested to be beneficial [128,133].

Treatment phase III is aimed at the maintenance and expansion of immune protection, and is considered vital to keep the ever-changing tumor from flaring up. The tumor immune escape mechanism is a well-described phenomenon [26,134]. By combining ICD immunotherapy, peptide vaccines targeting more universally spread tumor antigens like WT1 [135] and survivin [136], but also specific antigens, like IDH1R132H [98] or H3 K27M [137], and booster DC vaccines, the immune system is repetitively trained against both current and newly emerging tumor antigens, targeting the cancer cells directly and indirectly from the outside and from within. Protection is updated and expanded, which is crucial to create long-term control over the malignant glioma. At this stage, the exhaustion of T cells might be overcome by using anti-PD1 and anti-CTLA4 checkpoint inhibitors. The question has risen whether recommended doses should be kept, or lower doses can be used [138,139]. Also, at this stage, TME treatment with repurposed drugs remains essential.

The potential effectiveness of this multiphase combined treatment strategy, which includes different modes of immunotherapy, has been reported. Overall survival data were compared to data of control arms of reported contemporary randomized clinical trials, outlining the current expected overall survival of these patients [26,140]. Though the clinical risk profiles of the reported real world patients were comparable or worse than the patient profiles in the randomized clinical trials, use of the proposed multiphase combined treatment strategy (Figure 9) resulted in a relevant increase in the 2 year overall survival in the adult population [26,140].

## 5. Optimizing Therapy Requires Careful Consideration and Constant Monitoring

The complexity of GBM treatment has been clearly established, and is reflected in its many possible treatment strategies. It is vital to consider that all aforementioned therapies do not function as a monotherapy, but rather need thoughtful combination or supplementation to effectively combat GBM. Combination therapies must be considered carefully, as some treatments have opposing effects or cancel each other out. To administer immunotherapy during chemo- or radiotherapy would defeat the purpose of the immunotherapy, as CTx and RCT both specifically target immune cells besides tumor cells [26,133]. Another example comprises the application of checkpoint inhibitors: in a randomized phase II trial and biomarker study, the PD1-checkpoint inhibitor pembrolizumab was administered alone (*n* = 30) or in combination with the anti-angiogenesis agent bevacizumab (*n* = 50) to patients with recurrent GBM [50]. Despite ample pre-clinical and clinical support that dual VEGF and immune checkpoint blockades might enhance anti-tumor immune responses, the authors reported no benefit of combination therapy. Their explanations for this lack of effect include that a potential complementary benefit is dependent on tumor context, and might not work for GBM specifically. A second reason given includes their use of a high bevacizumab dosage; lower dosing of antiangiogenics is related to vasculature normalization, while high doses are reported to augment hypoxia within the tumor, thus worsening immunosuppression. Antiangiogenics may also decrease the intratumoral penetration of therapeutic antibodies [50]. As anti-PD1 therapy is reported to trigger infiltration and activation of tumor-infiltrating lymphocytes in the TME, therapy timing might also play a major role [141].

Unfortunately, tumors have developed many ways of immunotherapy resistance. How the adaptive metabolism in the tumor and its TME affects immune cells through the expression of immunoregulatory factors was discussed in subchapters 2.4 and 2.5, but it was only recently discovered how metabolic restrictions, imposed by the TME, facilitate immunotherapy resistance and should thus be considered in both the development and application of immunotherapeutic strategies. It was found that specifically targeting metabolic deficiencies, such as hypoxia or the generation of suppressive metabolites, are promising in combination with GBM SoC [142].

The tumor-inherent mechanism of antigen escape is one of the main reasons immunotherapies such as chimeric antigen receptor (CAR)-T cells do not work on solid tumors, as CAR-T cells depend on recognition of surface antigens [143]. There exist seven different mechanisms of antigen escape: (1) pre-existing target-negative tumor clones, in which tumor cells with absent or dim antigen expression survive a first immunotherapy and then rise up to form a secondary tumor; (2) antigen gene mutations or alternative splicing, leading to loss or downregulation of antigens, or emergence of neoantigens against which the immunotherapy was not trained; (3) deficiencies in antigen processing, in which mutations in CD81 expression or CD19 regulation lead to an absence of CD19 in B cells required to recognize antigens; (4) antigen redistribution, in which antigens are transferred from the cell membrane surface to subcellular locations; (5) lineage switch, mainly described in leukemia patients where a lymphoid tumor transforms into a myeloid phenotype to escape treatment; (6) epitope masking, which occurs when tumor cells contaminate the harvested T cells and lead to CAR molecules which bind to CD19 on tumor cells and mask them; and (7) trogocytosis-mediated antigen loss, when cells acquire plasma from other cells, which can facilitate the transfer of antigens and lead to reduced antigen expression in tumor cells [143]. To overcome tumor immunotherapy resistance, it is deemed vital to understand the TME and combine different immunotherapies to target the tumor in different ways and so combat its heterogeneity.

Another good example of the importance of well-considered combination therapy is THC treatment. Delta-9-tetrahydrocannabinol (THC) is a well-studied cannabinoid prescribed to alleviate the side effects of cancer treatment: loss of appetite, nausea, vomiting, pain, stress, and sleeplessness. THC is thought to have direct anticancer effects, promoting apoptosis in a Cannabinoid receptor-dependent manner, anti-angiogenesis, inhibiting proliferation and migration, and inducing cell cycle arrest [144]. Simultaneously, the use of THC might also have tumorigenic effects; the treatment of GBM U73-MG cell lines with THC resulted in increased cell proliferation [145]. In addition, use of THC is under suspicion of impeding immunotherapy. THC blocks JAK1 of the JAK-STAT pathway through cannabis receptor 2 (CNR2), which also blocks CD8+ T cell proliferation and anti-tumor immunity [146]. In an extensive retrospective study, cannabis use negatively impacted tumor response rate to nivolumab in 51 patients who used it in combination, as compared to 89 patients who solely used nivolumab [147], and was associated with a poorer clinical outcome [148]. Use of this ancillary drug during the first anticancer phase can be recommended, but in combination with immunotherapy is thus advised against [144,146,147,148].

There is an additional great potential in newly developed therapies, such as nucleic acid therapies. Nucleic acid therapies, such as mRNA, siRNA, miRNA, aptamers, circRNAs oligonucleotides, DNA, peptide nucleic acids, or cancer gene therapy, might be able to overcome hurdles that current immunotherapies cannot [149]. Initially explored for gene therapy, gene knockdown, and protein replacement, nucleic acid therapeutics have had promising results for hepatoblastoma, nephroblastoma, neuroblastoma, osteosarcoma, medulloblastoma, retinoblastoma, acute lymphoblastic leukemia and GBM [142]. Now, nucleic acids are being explored as immunotherapeutics for cancer. Nucleic acids are highly target-specific, small enough to cross the BBB, and they can directly affect the tumor or TME, or (re-)sensitize the malignancy to other combination therapies [150]. Patient-derived nucleic acids can easily be amplified in vitro, enabling therapy development from only small biopsy samples, and can be stored relatively well, making semi-off-the-shelf production possible. While DNA is more inexpensive and stable than RNA, it risks patient genome integration. On the other hand, nucleic acids need an efficient delivery method to avoid degradation by ubiquitous nucleases or triggering a local or systemic immune response. Nucleic acids can also cause off-target effects, have poor accumulation at tumor sites, and have a low blood stream circulation. To avoid this, delivery systems are under development [150].

This review is written in 2024/early 2025, and takes into account the currently available repurposed drugs that receive attention by the neuro-oncology community, for approaching five important hallmarks. It should be strongly acknowledged that other hallmarks are also focuses of novel treatment approaches. Particularly in the domain of malignant glioma, the hallmark of non-mutational epigenetic reprogramming is emerging as a targetable hallmark [151]. The gain of function proteins like mutant IDH or H3 can be approached. Vorasidenib, approved by the FDA for grade II astrocytoma [152], is already debated for its potential use in higher grade astrocytoma [153]. The efficacy of peptide vaccines against mutant IDH has been proven [98]. Targeting the gain-of-function mutants such as H3 K27M and H3 G34R remains challenging. Nevertheless, “epigenetic” therapies with HDAC inhibitors, DNA methylation inhibitors, and EZH2 inhibitors are still under development [154,155].

Though we offer a comprehensive overview over many factors that influence specific GBM mechanics, their interplay amongst themselves and the TME, there are still many other external influences which should be taken into account. In this review, we did not take external effects, such as family history, lifestyle habits or exposure to carcinogenic environmental factors into account. We excluded the microbiome from contemplation, though its importance is emerging [156,157]. On the topic of person-based effects, it must be noted that women possess enriched immunological signatures compared to men [158], which translates into a statistically significant 1 year survival advantage in female GBM patients. This effect was even more pronounced under the application of vaccine-based immunotherapy. Male GBM patients have a larger population of exhausted T cells [159]. To the best of our knowledge, the age at which the differences between sexes develop has not yet been researched. Beyond these factors that were not discussed, it must be taken into account that despite all we now know and learn about GBM and other brain malignancies using novel techniques, there is still much to discover.

One way to do this is by introducing more structured monitoring strategies. Current GBM and general cancer monitoring consist mainly of analytical snapshots. When a therapeutic plan is based only on a one-time assay, for instance on a pathological analysis after tumor resection, it ignores the many dynamic, swift changes in malignant glioma and its TME here outlined, often accompanied by treatment resistance. To actually enable proper adaptable treatment individualization, tumor and TME dynamics must be taken into account. Thus, we argue that tumor status analysis should not only commence with diagnostic procedures, but should be repeated regularly, in a similar way imaging and toxicity monitoring are. By monitoring the tumor as well as the patient and tumor immune state, cancer evolution can be detected and corrected earlier. Potential signs of treatment resistance can be caught and combatted with different therapeutic options, and a fast and accurate response is of vital importance. A treatment combination could be performed better when informed towards best effectivity. Moreover, continuous monitoring of GBM development would offer much more, and much more accurate, information to further improve GBM-treatment.

Liquid biopsy (LB) is a promising tool toward minimally invasive repeated monitoring of specific markers for disease and TME over the course of the disease. Distant from the original tumor, cell-free nucleic acids (cfDNA/RNA), extracellular vesicles (EVs) or circulating tumor cells (CTCs) can be harvested from blood, CSF, or urine. Several techniques to analyze samples derived from LB at time of diagnosis are commercially available [160,161,162,163,164,165,166,167]. Research programs under development or in clinical studies more often include LB, and are designed to evaluate the relevance of LB-monitoring in cancer evolution, therapy response and/or the onset of therapy resistance [168,169]. For over a century, GBM were classified only by their histological hallmarks: primary GBM development was considered to appear with no detectable precursors. Secondary tumors appeared to be so different from primary tumors, bearing mutually exclusive gene alterations, that they were considered two completely different tumor entities rather than a primary and secondary malignancy [170]. Molecular characterization through LB can support in GBM understanding: a current insight into actionable mutations or copy number aberrations of the tumor status can be obtained, and treatment can be adjusted accordingly [170]. However, the BBB poses a major obstacle. Rather than using non- or minimally invasive samples for LB, such as urine or blood, ctDNA must be harvested from CSF to obtain a high enough concentration for accurate analysis. By the time information can be obtained from blood samples, it is a clear indication of BBB breaking down [171], which unfortunately means treatment adjustments are occurring at the time of already increased aggressiveness in the tumoral process. Beyond the low concentration, the absence of brain tumor markers poses another challenge. Commercial CTC systems rely on epithelial tumor markers for tumor cell identification, which are absent in brain tumors. Research currently includes H3 K27M [172,173], glial fibrillary acidic protein GFAP [165], or a mixture of SOX2, Tubulin beta-3, EGFR, A2B5, and c-MET [174], but with mixed success. More research is needed to improve LB of malignant glioma as a monitoring tool. However, new attempts are promising. One interesting approach is the measurement of GBM-derived EVs in the plasma as biomarkers for diagnosis, prognosis and monitoring [175]. Especially in the context of immunotherapy, longitudinal sampling of biofluids for multi-omics to dissect complex temporal changes in the GBM TME as a function of the immunotherapy is recommended [176].

Lastly, a multiphase combined treatment strategy is hypothesized and was tested against randomized clinical trials data. It is known that patients considered eligible for randomized clinical trials represent a thoroughly selected minority of patients in a real-world population [177]. Nonetheless, the application of the proposed multiphase combined treatment strategy with real world patients resulted in a relevant improvement of overall survival when compared to clinical trials [26,140]. It will be challenging to translate multiphase individualized combination treatment strategies into appropriate clinical trial strategies, so that the treatment approach becomes reproducible, for the benefit of all patients [19,177].

The implementation of individualized therapy does come with its own unique challenges. Though therapy options are improving, they often remain highly expensive due to their patient-specific nature. Often, individualized cell therapies cannot be prepared off-the-shelf, which means the need for a specific clean-room, specific equipment, trained staff, and GMP-certified processes within treatment facilities. Adaptation of personalized treatment would require a major rethink on treatment validation, as clinical trials usually require a standardized protocol in a standardized group of patients, both of which cannot apply [19]. These aspects complicate individualized therapy. However, it has also been shown that personalized treatment shortens the actual hospital or clinic stay, and vastly improves patient quality of life. Especially for GBM, the most frequent primary brain tumor in adults with the highest mean years of potential life lost amongst all human cancers [5], there is much to be gained.

Similarly, the use of repurposed drugs in GBM treatment should be considered carefully. It is often considered a fast-track in GBM treatment. As these drugs have already been applied to solve other medical problems, they often have been extensively tested for safety and pharmacokinetic properties. Repurposed drugs are often more readily available and relatively cheap compared to alternatives that may not yet be available [178,179]. However, the BBB can limit the ability of these drugs to reach the tumor site. Severe side effects can be expected when dealing with repurposed drugs. It must also be validated that the drugs can actually reach and affect the tumor [178,179].

## 6. Conclusions

In this review, we have discussed five tumor hallmarks. We outlined how they develop, what their molecular and cellular effects are, how they are affected by the TME and how they respond to treatment. It is clear that metabolic activity, nutrient availability, extracellular communication and hypoxia are cancer hallmarks which cause and contribute to the highly heterogeneous, spatio-temporal fluctuating, fast-adapting character of GBM and its TME. An in-depth analysis of the many possible processes happening simultaneously in cancer intracellular maintenance and progression makes it clear that GBM functions as a complex regulated system rather than a simple cluster of independent fast-growing cells. The treatment responses and options listed indicate how multi-faceted treatment should be, in order to not only keep up with the tumor heterogeneity but to get ahead of it. To be able to effectively target the tumor as a whole, a thorough understanding of the cancer and its immediate surrounding tissue is required [140]. Several categories of repurposed drugs, and several modes of immunotherapy in combination with standard of care (SoC) are considered vital to treat not only the heterogeneous tumor but the specific GBM hallmarks. We believe it is vital to document and consider tumor location, metastases, primary or secondary, age, sex, MGMT promoter methylation status, mutations present or developing, and macrophage population, as these malignancy characteristics have a major influence on treatment strategy and response, and should be considered. However, initial documentation and consideration are not enough to treat an ever-changing tumor. We deem it critical to incorporate repeated monitoring. To properly combat the spatio-temporal aspects of GBM and its TME, repeated monitoring, for instance through liquid biopsy, of the tumor status and its development is necessary to ensure the treatment plan matches the actual tumorigenic aspects.

Further research will give more insight into other tumor hallmarks, and may lead to the development of accurate GBM subtype development models. Until that time, we depend on current patient information and treatment flexibility. Currently, tumor tissue from a first resection is often analyzed to determine post-surgery treatment. Primary in each treatment plan should be the extermination of GSCs, as these are highly treatment-resistant while offering GBM much of its heterogeneity. Antidepressants can be used against GSCs, and the TME must be carefully monitored and treated to avoid new GSCs developing. An individualized, multi-faceted, and multi-phased treatment plan should support the current SoC. With all the new developments in tumor understanding, tumor treatment cannot be left behind with only currently available SoC.

## Abbreviations

The following abbreviations are used in this manuscript:

TME	Tumor microenvironment
GSCs	Glioma stem-like cells
CNS	Central Nervous System
GBM	Glioblastoma
Tregs	T regulatory cells
NK	Natural Killer cells
GAMs	Glioma- or Tumor-associated microglia and macrophages, also known as TAMs
MDSC	Myeloid-derived suppressor cells
TAN	Tumor-associated neutrophils
BBB	Blood–brain barrier
SoC	Standard-of-Care
RCT	Radiochemotherapy
RT	Radiotherapy
CTx	Chemotherapy
Sc-seq	Single cell sequencing
miRNA	Micro-RNA
GTMEI	GBM-associated TIME immune cell infiltration
WHO	World Health Organization
Tcm	Central memory T cells
Tfh	T follicular helper cells
TMZ	Temozolomide
EMT	Epithelial-to-mesenchymal transition
VEGF	Vascular endothelial growth factor
EPCs	Endothelial progenitor cells
HIF	Hypoxia-inducible factor
CSF	Cerebrospinal fluid
LLMs	Lipid-laden macrophages
HMGB	high mobility growth factor
PDK	pyruvate dehydrogenase kinase
IL	Interleukin
ROS	Reactive oxygen species
OXPHOS	Oxidative phosphorylation
ACSS2	acetyl-CoA synthase
TGF	transforming growth factor
NO	Nitric oxide
FU	Fluorouracil
MMP	matrix metalloproteinases
CITE-seq	cellular indexing of transcriptomes and epitomes by sequencing
CyTOF	cytometry by time of flight
ER	endoplasmic reticulum
CPM	Cyclophosphamide
NDV	Newcastle Disease Virus
DMG	Diffuse midline gliomas
DIPG	diffuse intrinsic pontine glioma
pDHGG	diffuse pediatric-type high-grade glioma
H3 G34-mutant DHG	diffuse hemispheric glioma H3G34-mutant
H3 K27-altered DMG	diffuse midline glioma H3K27-altered
MRS	magnetic resonance spectroscopy
MRI	magnetic resonance imaging
DRD2	dopamine receptor D2
ClpP	mitochondrial caseinolytic protease P
α-KG	α-ketoglutaric acid
TNT	tunneling nanotubes
ICD	immunogenic cell death
mEHT	modulated electrohyperthermia
TTF	Tumor Treating Fields
DCs	Dendritic cells
LB	Liquid biopsy
EVs	extracellular vesicles
CTCs	circulating tumor cells
CAR-T cells	chimeric antigen receptor T cells
